# Accumulation of neurofibrillary tangles and activated microglia is associated with lower neuron densities in the aphasic variant of Alzheimer’s disease

**DOI:** 10.1111/bpa.12902

**Published:** 2020-11-05

**Authors:** Daniel T. Ohm, Angela J. Fought, Adam Martersteck, Christina Coventry, Jaiashre Sridhar, Tamar Gefen, Sandra Weintraub, Eileen Bigio, M.-Marsel Mesulam, Emily Rogalski, Changiz Geula

**Affiliations:** 1 Mesulam Center for Cognitive Neurology and Alzheimer’s Disease, Northwestern University Feinberg School of Medicine, Chicago, IL.; 2 Department of Preventive Medicine, Northwestern University Feinberg School of Medicine, Chicago, IL.; 3 Department of Psychiatry and Behavioral Sciences, Northwestern University Feinberg School of Medicine, Chicago, IL.; 4 Department of Pathology, Northwestern University Feinberg School of Medicine, Chicago, IL.; 5 Department of Neurology, Northwestern University Feinberg School of Medicine, Chicago, IL.

**Keywords:** activated microglia, Alzheimer’s disease, neurons, neurofibrillary tangles, primary progressive aphasia

## Abstract

The neurofibrillary tangles (NFT) and amyloid-ß plaques (AP) that comprise Alzheimer’s disease (AD) neuropathology are associated with neurodegeneration and microglial activation. Activated microglia exist on a dynamic spectrum of morphologic subtypes that include resting, surveillant microglia capable of converting to activated, hypertrophic microglia closely linked to neuroinflammatory processes and AD neuropathology in amnestic AD. However, quantitative analyses of microglial subtypes and neurons are lacking in non-amnestic clinical AD variants, including primary progressive aphasia (PPA-AD). PPA-AD is a language disorder characterized by cortical atrophy and NFT densities concentrated to the language-dominant hemisphere. Here, a stereologic investigation of five PPA-AD participants determined the densities and distributions of neurons and microglial subtypes to examine how cellular changes relate to AD neuropathology and may contribute to cortical atrophy. Adjacent series of sections were immunostained for neurons (NeuN) and microglia (HLA-DR) from bilateral language and non-language regions where *in vivo* cortical atrophy and Thioflavin-S-positive APs and NFTs were previously quantified. NeuN-positive neurons and morphologic subtypes of HLA-DR-positive microglia (i.e., resting [ramified] microglia and activated [hypertrophic] microglia) were quantified using unbiased stereology. Relationships between neurons, microglia, AD neuropathology, and cortical atrophy were determined using linear mixed models. NFT densities were positively associated with hypertrophic microglia densities (*P* < 0.01) and inversely related to neuron densities (*P* = 0.01). Hypertrophic microglia densities were inversely related to densities of neurons (*P* < 0.01) and ramified microglia (*P* < 0.01). Ramified microglia densities were positively associated with neuron densities (*P* = 0.02) and inversely related to cortical atrophy (*P* = 0.03). Our findings provide converging evidence of divergent roles for microglial subtypes in patterns of neurodegeneration, which includes hypertrophic microglia likely driving a neuroinflammatory response more sensitive to NFTs than APs in PPA-AD. Moreover, the accumulation of both NFTs and activated hypertrophic microglia in association with low neuron densities suggest they may collectively contribute to focal neurodegeneration characteristic of PPA-AD.

## INTRODUCTION

Intracellular neurofibrillary tau tangles (NFTs) and extracellular amyloid-ß plaques (APs) comprise the pathologic hallmarks of Alzheimer’s disease (AD). NFTs and APs cause patterns of neurodegeneration marked by cellular changes that remain poorly understood. A common response to AD neuropathology is glia activation, which includes microglial activation associated with neuroinflammatory processes that may play an important role in disease pathogenesis and severity in those with amnestic AD (49, 63, 75). The complex microglial response involves microglia adopting a range of morphologic subtypes to maintain cellular homeostasis (31), with some subtypes better recognized and closely linked to neurodegenerative processes compared to others. For example, resting microglia often have a *ramified* appearance given their relatively greater number of longer and thinner processes that likely survey their surroundings for pathophysiological changes that can trigger a conversion to an activated state (55, 61, 86). Activated microglia are the brain macrophages that react and co-localize with AD neuropathology and classically appear *hypertrophic* based on thick and short processes reflecting increased production of cytokines, surface-antigen expression, and growth factors associated with neuroinflammation (36, 48, 55, 68, 74, 75, 77). Investigating microglial subtypes in the context of neuropathologic changes is important to the ongoing effort to characterize the phenotypic and functional heterogeneity of microglia in health, aging, and disease (10, 23, 30, 58).

Neuronal changes associated with AD neuropathology include reductions in axonal density (19, 78), extent of dendrites (3, 15), and number of synapses (2, 70, 85). Neurons also die by the end stage of disease and their loss correlates with NFT number (28), but the extent of reported neuronal loss has been inconsistent. Neuronal degeneration may also be a major substrate of *in vivo* cortical atrophy, but the relationship between neurons and atrophy is not well understood outside of small regions such as the hippocampus in amnestic AD (90). Quantitative investigations of neuronal and microglial changes outside of individuals with amnestic AD remain sparse, specifically in non-amnestic clinical variants of AD (9, 60).

The aphasic variant of AD, also known as primary progressive aphasia caused by AD neuropathology (PPA-AD) (1, 26, 40, 50, 53, 66, 69, 87), is a language dementia syndrome characterized by an asymmetric pattern of cortical atrophy concentrated to the language dominant hemisphere (51, 64, 65, 67). Unlike the symmetric distributions of APs and NFTs typically observed in amnestic AD (57), NFTs have higher densities in left language regions, displaying concordance with the cortical atrophy patterns of PPA (26, 37, 59). Given that NFTs are a major neuropathologic determinant of *in vivo* cortical atrophy in the amnestic and aphasic presentations of AD (17, 59), the atypical deposition of AD neuropathology in PPA raises the question of whether neurons and activated microglia also form atypical distributions in PPA-AD. The focal, asymmetric profile characteristic of PPA provides a useful model to test the hypothesis that neuronal loss and increased microglial activation correspond to the known patterns of NFT burden and cortical atrophy unique to PPA-AD.

The primary aim of the current study of PPA-AD was to acquire stereologic estimates of neurons and microglial subtypes in the gray matter to determine if densities of neurons and microglia reflect the accumulation of AD neuropathology quantified previously (59). To determine gray and white matter relationships rarely reported in postmortem studies, pathologic and cellular markers quantified in gray matter were compared to optical densities of activated microglia measured previously (60) in adjacent white matter in a subset of available regions in PPA-AD. Finally, we examined the cellular basis of *in vivo* cortical atrophy in PPA-AD by comparing densities of neurons and microglia to measurements of atrophy in matching regions. Regions of interest included language and non-language areas in the left language-dominant hemisphere in addition to their contralateral homologues. Given that hypertrophic microglia (HM) are the morphologic subtype more frequently implicated in neuroinflammation and disease compared to ramified microglia (RM), it was hypothesized that HM would be associated with cortical atrophy and greater densities of NFTs, APs, and white matter activated microglia. Lower densities of neurons were also anticipated in regions with greater densities of AD neuropathology, HM, and cortical atrophy.

## MATERIALS AND METHODS

### Participants

The current study enrolled participants through the PPA Research Program at the Mesulam Center for Cognitive Neurology and Alzheimer’s disease at the Northwestern University Feinberg School of Medicine. As described previously (59, 60), the participants included five right-handed individuals who received clinical diagnoses of PPA (29, 51, 52) and pathologic diagnoses of “high” AD neuropathologic change based on published consensus criteria (35, 56). Two PPA participants presented with features consistent with the logopenic variant of PPA, one with the agrammatic variant, and two were unclassifiable. Importantly, each PPA participant had acquired a structural MRI scan within 2.5 years of death that was used to measure cortical atrophy relative to a previously described cognitively healthy control group (59, 64) consisting of 35 participants of similar age (PPA group: median 62 years, range 59–76 years; control group: median 62 years, range 50–74 years; Wilcoxon rank-sum *P* = 0.23) and education (PPA group: median 15 years, range 12–18 years; control group: median 16 years, range 11–20 years; Wilcoxon rank-sum *P* = 0.38). The Northwestern University Institutional Review Board approved this study and all participants gave consent to their involvement and brain donations. Characteristics of each PPA participant are summarized in Table 1.

### MRI acquisition

All PPA and healthy control participants had structural MRI scans acquired on a 3T Siemens TIM Trio scanner using a 12-channel birdcage head coil at the Northwestern University Center for Translational Imaging. A T_1_-weighted 3D MPRAGE sequence included the following: repetition time = 2300 ms, echo time = 2.91 ms, inversion time = 900 ms, field of view = 256 mm, flip angle = 9°, 1 mm^3^ voxel resolution collected over 176 sagittal slices. The scan-to-death mean interval was 1.96 ± 0.8 years; range 0.61–2.54 years (Table 1).

### MRI analysis and regions of interest

**Figure 1.**
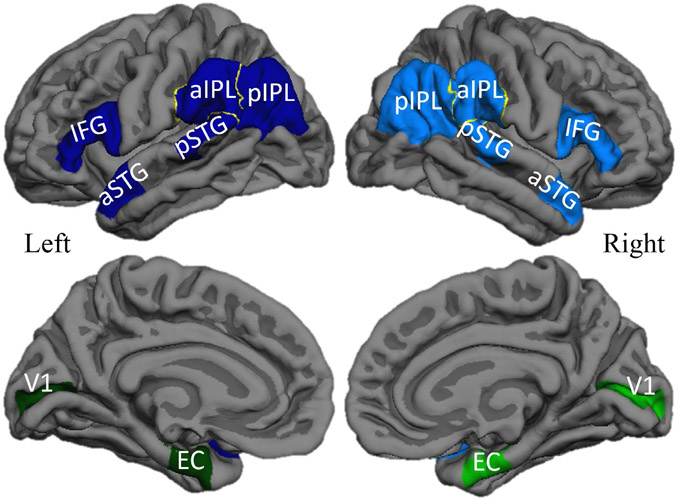
Regions of interest in PPA-AD. Left language ROIs (dark blue), right language homologues (blue), left non-language ROIs (dark green), right non-language ROIs (green) displayed bilaterally on lateral (top) and medial (bottom) reconstructions of a template cortical surface in FreeSurfer. Abbreviations: IFG = inferior frontal gyrus; aSTG = anterior superior temporal gyrus; pSTG = posterior superior temporal gyrus; aIPL = anterior inferior parietal lobule; pIPL = posterior inferior parietal lobule; EC = entorhinal cortex; V1 = primary visual cortex.

Structural MRI scans were processed according to our previous description (59) using FreeSurfer (v5.1.0, http://surfer.nmr.mgh.harvard.edu) with manual correction of topological surface errors according to established guidelines (22, 72). In brief, regional cortical atrophy was determined by comparing the five PPA participants to 35 cognitively healthy controls with similar demographics. The last MRI scan before death was used for each PPA participant. FreeSurfer was used to measure the mean cortical thickness in identical regions of interest (ROIs) created and used previously (59) that included 14 ROIs in each PPA participant, 7 per hemisphere (Figure 1). The left language-dominant hemisphere contained five language-related and two non-language ROIs. The right hemisphere contained the seven homologues of the left hemisphere ROIs. The “language ROIs” consisted of the inferior frontal gyrus (IFG), anterior superior temporal gyrus (aSTG), posterior superior temporal gyrus (pSTG), anterior inferior parietal lobule (aIPL), and posterior inferior parietal lobule (pIPL). The bilateral “non-language ROIs” were the memory-related entorhinal cortex (EC) and the primary visual cortex (V1). The mean cortical thickness of each ROI in each participant was converted to a standardized *z*-score (representing the magnitude of cortical atrophy in each ROI) using the mean (*μ*) and standard deviation (*σ*) from the healthy control group:

$$z=(\mu_{\text{healthy controls}}-[\text{participant mean cortical thickness}])∕\sigma_{\text{healthy controls}}$$

### Tissue processing

Brains of PPA-AD participants were processed as previously described for staining of microglia (60) or AD neuropathology and neurons (59). Brains were cut into ~1–2 cm coronal blocks and fixed in either 10% formalin for 2 weeks or 4% paraformaldehyde for 30–36 hours at 4°C, and then, submerged in an increasing concentration gradient of sucrose (10%–40%) for cryoprotection (Table 1). Blocks containing ROIs were cut into 40 μm-thick coronal sections to collect three adjacent 1:24 series of postmortem sections for staining and quantitation of neurons, microglia, and AD neuropathology. Each postmortem series of tissue contained 5–16 sections depending on the anterior-posterior extent of each ROI defined by FreeSurfer and the thickness of the postmortem coronal block. AD neuropathology was visualized using the Thioflavin-S stain (1%), while microglia and neurons were visualized immunohistochemically without antigen retrieval using the avidin–biotin peroxidase complex method employing the Vectastain Elite Kit and 3,3-diaminobenzidine as the chromogen. Neurons were visualized using an antibody against neuronal nuclear protein (NeuN, mouse monoclonal; EMD Millipore; 1/2000) (88). The microglial subtypes of interest to the current investigation (i.e., HM and RM) were consistently detected using an antibody against the human leukocyte antigen-D related protein (HLA-DR, mouse monoclonal; Dako; 1/1000), a class II cell-surface glycoprotein of the major histocompatibility complex (27, 47, 48, 82). Immunostaining for HLA-DR in thick postmortem sections permitted the reliable visualization of the extensive arbor and diverse morphologies characteristic of microglia. HLA-DR-positive microglia that met morphologic criteria for distinct microglial subtypes are described below. The left/right hemispheric pair of each region was always stained together in the same immunohistochemical run for each marker of interest.

### ROI correspondence

Anatomical correspondence between *in vivo* MRI and postmortem tissue was achieved using methods described previously (59). In brief, all series of postmortem tissue were collected from MRI-based ROIs generated a priori in FreeSurfer. Postmortem regional boundaries were delineated on every section of NeuN-positive tissue that matched MRI ROI boundaries. Given that NeuN-positive tissue was a parallel series to the HLA-DR-positive and Thioflavin-S-positive tissue, it provided the means to transfer the same regional boundaries onto adjacent sections stained for HLA-DR or Thioflavin-S. This ensured that all postmortem data corresponded to each other and to measures of *in vivo* cortical atrophy.

White matter activated microglia were previously quantified (60) in a subset of the 14 ROIs used for stereologic investigations in the present study. The subset included two language and two non-language ROIs in the language dominant hemisphere and their contralateral homologues for a total of eight ROIs examined per PPA participant. The non-language ROIs were always V1 and EC, two regions shown to have less AD neuropathology compared to language ROIs in PPA-AD (26, 59). Language ROIs were selected based on peak cortical atrophy in each PPA participant and thus, varied by participant (60) (Table 2). The current investigation performed stereology on the same coronal sections per ROI where white matter activated microglia were assessed to establish gray and white matter relationships rarely examined postmortem in neurodegenerative diseases.

### Stereologic quantification

Densities of neurons, HM, and RM were quantified at a final magnification of 60× across all cortical layers of each ROI using an unbiased stereologic analysis. Morphologic subtypes of microglia (i.e., HM and RM) were counted in tandem using the following criteria clearly identifiable within the depth of tissue: HM were defined as having darker immunoreactivity for HLA-DR throughout their enlarged somas, and thicker, shorter processes relative to RM, which were defined as having lighter immunoreactivity for HLA-DR throughout their smaller somas and thinner, more branched arbor. Dystrophic microglia were infrequent and unreliably discernible and therefore, not included in this stereologic investigation of HLA-DR immunoreactive microglia. Overlapping, closely packed microglia were also infrequent and excluded from analysis because it precluded a reliable differentiation of individual microglia for analysis. Stereologic estimates of NFT and dense-core AP densities were acquired previously in the same regions as neurons and microglia (59). All analyses were performed on a workstation equipped with a Nikon Eclipse E800 microscope, motorized stage, and stereology software (Stereo Investigator v11.07, MBF Bioscience). The optical fractionator probe was used to estimate the populations of each cellular marker from all available sections per ROI, with sampling grid dimensions that varied by anatomical region to produce a coefficient of error ≤0.1 (71). The size of the counting frame was kept at 125 μm^2^, and a dissector height of 16 μm with guard zones of 2 μm was used. Section thickness was measured at each counting site to calculate an average section thickness used in estimating populations of each cellular marker. For each ROI, densities of HM, RM, and neurons per mm^3^ were calculated by taking the estimated population using number weighted section thickness and dividing by the planimetry volume.

### Statistical analyses

All analyses were conducted using linear mixed models accounting for repeated measures. Given the main focus of the current investigation was to understand how AD neuropathology (NFT and AP densities) was related to cellular markers (HM, RM, white matter activated microglia, and neuron densities), the first set of models examined the relationships between two postmortem variables at a time. Given that none of the variables were obvious outcomes or predictors, we evaluated the relationships twice. For example, when comparing HM and RM, we ran two models including one with HM as the outcome and then a second with RM as the outcome, using a Bonferroni corrected significance level of 0.025. Only one model was reported, selecting the larger, more conservative *P* value of the two models. To evaluate the cellular basis of cortical atrophy, models examined the relationship between cortical atrophy (measured as *z*-scores) and each cellular marker (measured as densities). Models that examined the relationships with white matter activated microglia incorporated a subset of regions (Table 2). All models used in the first set of analyses were adjusted for hemisphere, type of region (language or non-language), and age at death. When cortical atrophy was compared to postmortem marker densities, models were also adjusted for scan-to-death interval and when postmortem markers were compared to each other, models were adjusted for postmortem interval.

Given our previous findings showing that NFTs and cortical atrophy are significantly greater in the left language regions of this PPA-AD cohort (59), a second set of analyses characterized the distributions of HM, RM, white matteractivated microglia, and neurons. To assess if each cellular marker displayed hemispheric asymmetry, the relationship between each cellular marker and hemisphere was evaluated after stratifying by language or non-language regions. To determine if cellular marker densities were different between language and non-language regions, the association between each cellular marker and type of region (language or non-language) was assessed in only the left hemisphere. All models used in the second set of analyses were adjusted for age at death and postmortem interval. Significance was set to *P* < 0.05 for all comparisons unless otherwise stated. Analyses were performed using SAS software (v9.4; SAS Institute).

## RESULTS

### Qualitative distributions of neurons, microglia, NFTs, and APs in language regions

Neurons, microglia, and neuropathology formed distinct distributions across regions and cortical layers in PPA-AD. A detailed cortical layer analysis was beyond the scope of the current quantitative investigation, but qualitative descriptions are reported here to provide further context to the novel quantitative relationships found in the full extent of gray matter (see below). Immunoreactivity did not vary in accordance with fixative type, fixative duration, postmortem interval, or any known preagonal states that could alter expression of proteins of interest.

**Figure 2.**
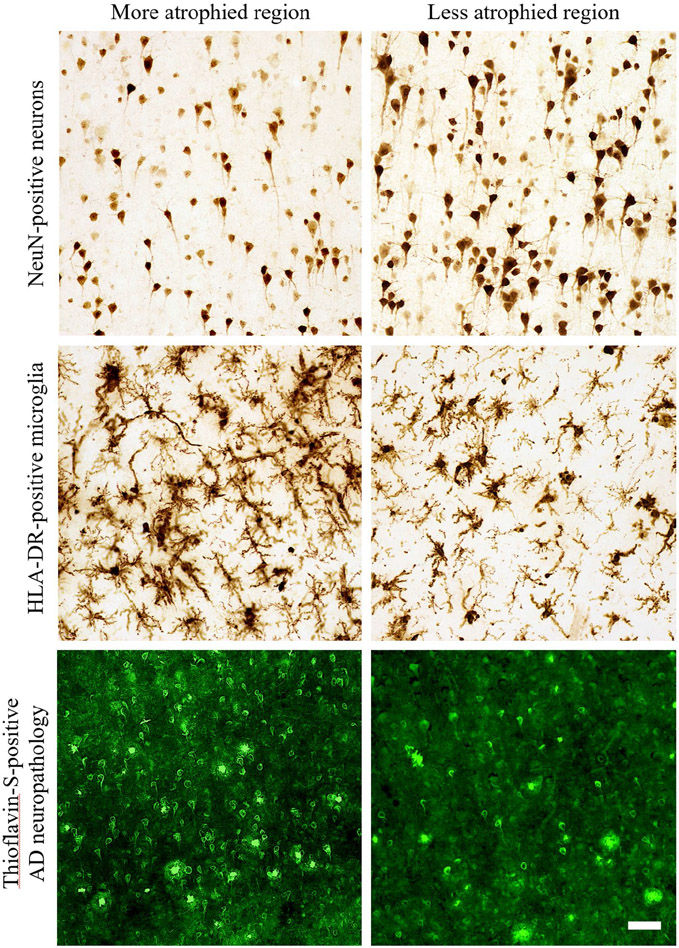
Representative photomicrographs of the differential distributions of neurons, activated microglia, and AD neuropathology in regions with high or low cortical atrophy in a PPA-AD participant. More atrophied regions often displayed lower densities of NeuN-positive neurons and higher densities of HLA-DR-positive activated microglia and Thioflavin-S-positive NFTs in comparison to less atrophied regions. Photomicrographs of each marker were acquired at 20× magnification and centered over cortical layer III within the gyral crown of bilateral aIPL in PPA-AD participant #3. More atrophied region—left aIPL; less atrophied region—right aIPL. Scale bar set to 50 μm.

NeuN-positive neurons were consistently less dense in layers III and V of language regions, with often the least NeuN immunoreactivity in layer III of highly atrophied regions (Figure 2, top panel centered over layer III bilaterally). It is noteworthy that the predominant output of layer III neurons is to neighboring cortical regions where they form the association U-fibers, the same area of white matter where more microglial activation was often observed compared to neighboring white matter tracts and layer VI (60). Reduced NeuN immunoreactivity (i.e., lower intensity of staining) and/or lower densities of neurons were occasionally observed toward the gyral fundus relative to the gyral crown. A similar pattern has been previously reported for NFTs and APs in amnestic AD such that more were observed in gyral fundi (4, 11).

Hypertrophic microglia, representing the predominant subtype of activated microglia (38, 55, 77, 79, 86), were more frequent than RM especially in atrophied gray matter regions (Figure 2, middle panel centered over layer III bilaterally). RM formed no recognizable pattern across cortical layers because of their lower densities. HM sometimes formed large, overlapping clusters that were more common across layers II-V, with lower densities in layer IV and very few, if any, in layers I and VI in all regions examined. As we have described previously (60), the majority of HLA-DR-positive microglia in the white matter were of the HM subtype, and thus, mostly activated microglia, with high, overlapping densities concentrated to U-fibers that precluded reliable stereologic assessment in white matter.

The distribution of gray matter HM is noteworthy given its similarity to the laminar distribution of dense-core APs and NFTs, as well as previously reported topographic patterns of activated microglial clusters/nests in amnestic AD or frontotemporal lobar degeneration (12, 43, 83, 84). More specifically, we observed NFTs more frequently in layers II, III, and V throughout language regions of PPA-AD (Figure 2, bottom panel centered over layer III bilaterally). Dense-core APs typically accumulated in layers II-V without obvious laminar selectivity. The laminar deposition of AD neuropathology in PPA-AD appeared consistent with what has been reported in cortical and limbic regions of amnestic AD (5, 11, 12, 33, 44, 62). Diffuse plaques were more frequent than dense-core APs, but did not display a recognizable pattern between cell layers, language regions, or hemispheres. Across all regions examined, layer I was consistently devoid of dense-core APs and NFTs, and the deepest part of layer VI immediately adjacent to white matter showed infrequent APs and nearly no NFTs. NFTs were exceedingly rare in cortical white matter, but diffuse plaques and dense-core APs were sometimes present in the superficial U-fibers of white matter, an observation that has been reported previously (73).

### Qualitative distributions of microglia, neurons, NFTs, and APs in non-language regions

In comparison to language regions, non-language regions displayed not only similar but also divergent patterns in cell marker distribution. HLA-DR immunoreactivity was generally more pronounced in the upper layers I-III compared to the lower layers IV-VI of EC. In V1, clusters of HM were concentrated in the stria of Gennari which also displayed stronger HLA-DR immunoreactivity than other cortical layers but less intense than surrounding white matter. Neuron density appeared greatest in layers II and VI of both EC and V1. NFTs were most prominent in layer II and common in layer V of EC, while virtually no NFTs were observed in any layers of V1. Dense-core APs were common across most layers of V1 and relatively rare in EC, while diffuse plaques were common in EC and scarce in V1. Whereas EC layer II stellate cell islands were readily discernible by large NFT-bearing neurons, the islands were inconsistently detected by densities of HM or NeuN-positive neurons.

### Quantitative distributions of microglial subtypes and neurons

**Figure 3.**
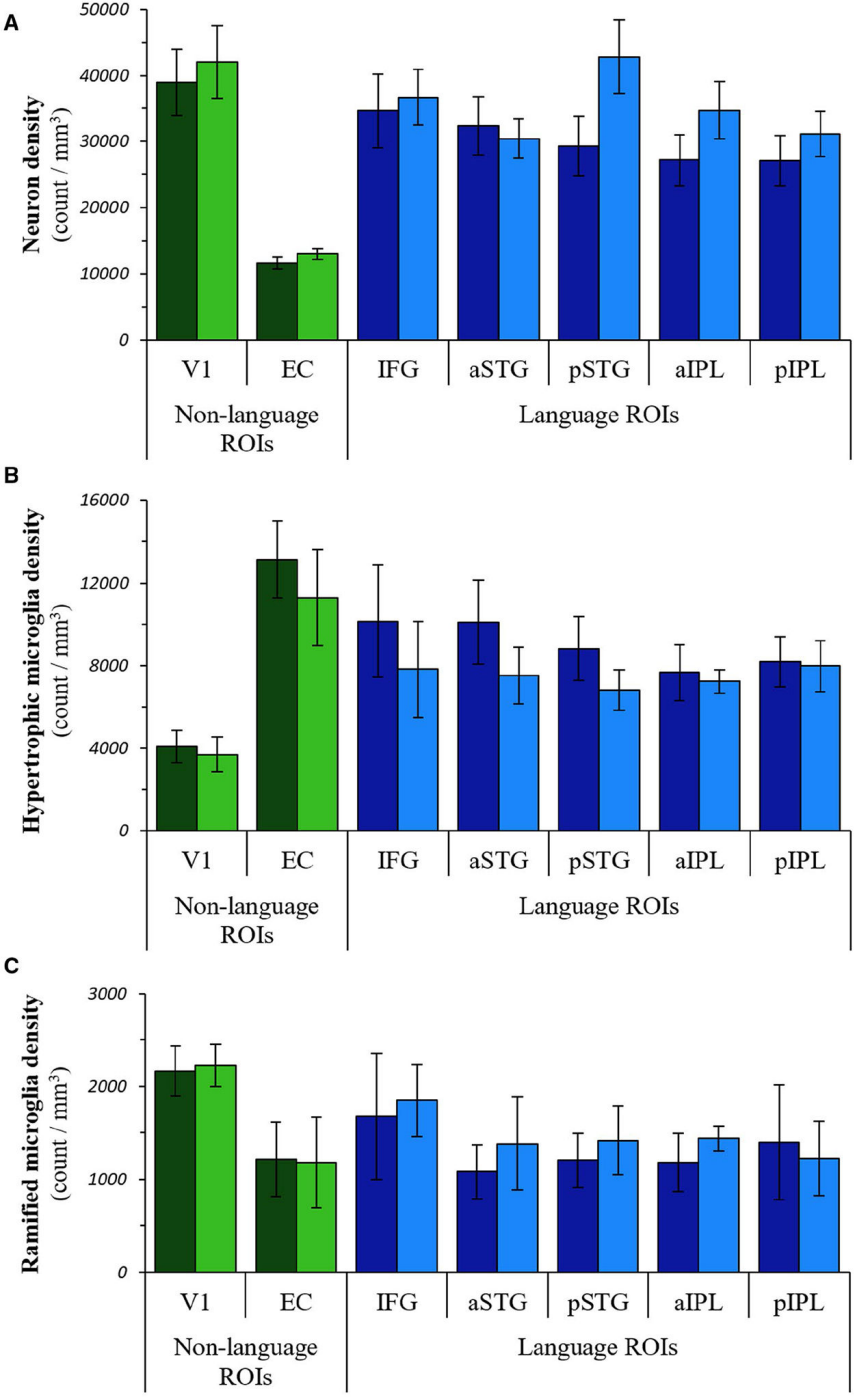
Distributions of neurons and microglial subtypes in PPA-AD. The regional distributions of neuron densities (**A**), hypertrophic microglia densities (**B**), and ramified microglia densities (**C**) in PPA-AD. Note that tests were not performed on individual regions. Left language ROIs = dark blue bars; right language homologues = blue bars; left non-language ROIs = dark green bars; right non-language ROIs = green bars.

Densities of HM, RM, and neurons did not display significant hemispheric asymmetry or language region selectivity (Figure 3, Tables S1 and S2). HM, however, did show greater densities in the left language regions (estimated mean density: 8988) compared to right homologues (estimated mean density: 7475) that trended toward significance (*P* = 0.08), (Table S1). Densities of RM and neurons were lower in the left language regions compared to right language homologues (RM estimated means: left 1309, right 1461; neuron estimated means: left 30 125, right 35 164), but these differences did not reach statistical significance (Tables S1 and S2).

### Relationships between microglia, neurons, NFTs, and APs

**Figure 4.**
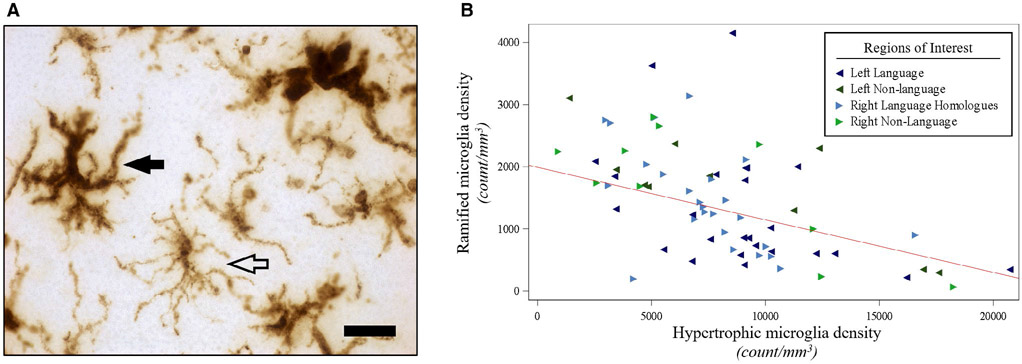
Hypertrophic microglia densities were negatively associated with ramified microglia densities in PPA-AD. (**A**) Representative photomicrograph of morphologic subtypes of HLA-DR-positive microglia. Hypertrophic microglia (filled arrow) displayed darker immunoreactivity for HLA-DR throughout their enlarged somas, and thicker, shorter processes relative to ramified microglia (open arrow), which displayed lighter immunoreactivity for HLA-DR throughout their smaller somas and thinner, more branched arbor. Photomicrograph acquired at 63× magnification. Scale bar set to 25 μm. (**B**) There was a negative relationship (*P* < 0.01) between ramified microglia densities and hypertrophic microglia densities, as shown in the predicted line (slope: −0.08) from the mixed model, using the left hemisphere, language regions, and the medians for age at death (64) and postmortem interval (14) for the adjusting variables. Data displayed include all 5 participants with 14 observations each (five language and two non-language regions per hemisphere).

Hypertrophic microglia densities in gray matter were inversely related to RM densities (*P* < 0.01), (Figure 4B, Table S3) and on average, HM densities were over sixfold greater than RM densities in each hemisphere. Mean optical densities of white matter activated microglia showed a positive association with gray matter HM densities (*P* < 0.01), and a negative association with gray matter RM densities (*P* < 0.01), (Table S3).

**Figure 5.**
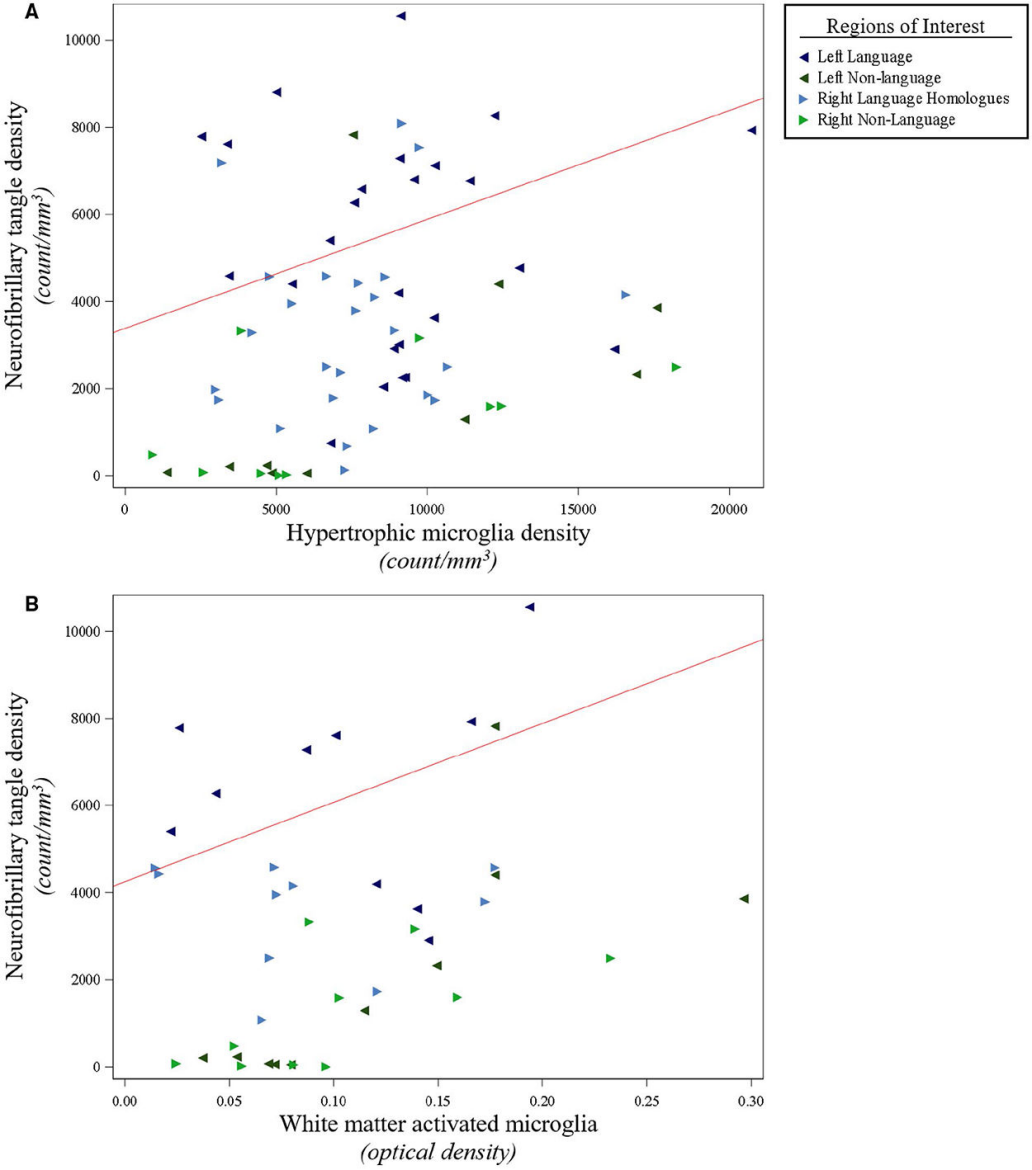
NFT densities were positively associated with activated microglia densities in gray matter and white matter of PPA-AD. (**A**) There was a positive relationship between NFT densities and activated hypertrophic microglia densities (*P* < 0.01), as displayed by the predicted line (slope: 0.25) from the mixed model. Data displayed include all 5 participants with 14 observations each (five language and two non-language regions per hemisphere). (**B**) There was a positive relationship between NFT densities and optical densities of white matter activated microglia (*P* < 0.01), as illustrated by the predicted line (slope: 18 212). Data displayed include all five participants with eight observations each (two language and two non-language regions per hemisphere). When creating the predicted lines, we used the left hemisphere, language regions, and the medians for age at death (64) and postmortem interval (14) for the adjusting variables.

**Figure 6.**
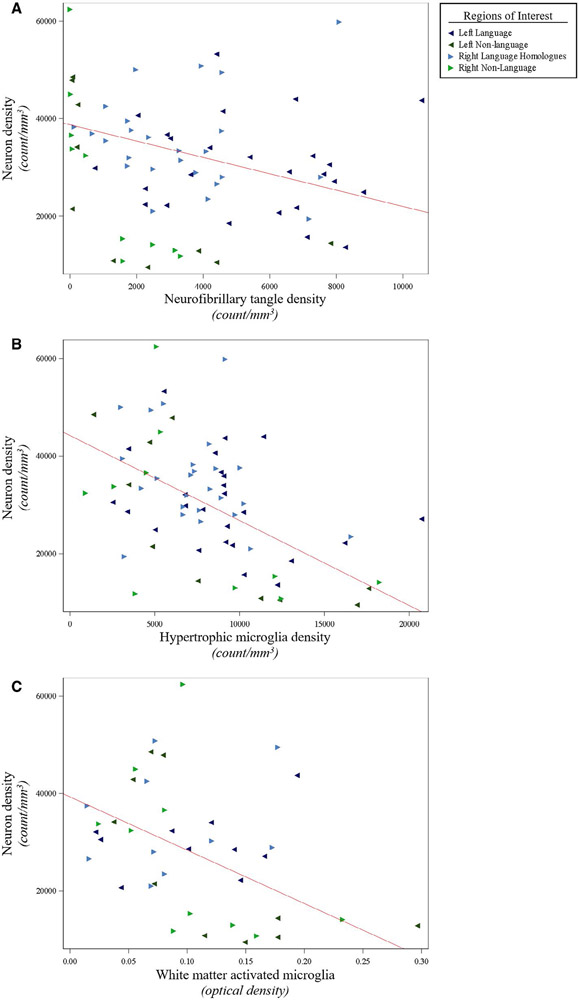
Smaller neuron densities were associated with larger densities of NFTs and activated microglia in PPA-AD. (**A**) There was a negative relationship between neuron densities and NFT densities (*P* = 0.01), as shown in the predicted line (slope: −1.68) from the mixed model. Data displayed include all 5 participants with 14 observations each (five language and two non-language regions per hemisphere). (**B**) There was a negative relationship between neuron densities and activated hypertrophic microglia densities (*P* < 0.01), as displayed by the predicted line (slope: −1.74) from the mixed model. Data displayed include all 5 participants with 14 observations each (five language and two non-language regions per hemisphere). (**C**) There was a negative relationship between neuron densities and optical densities of white matter activated microglia (*P* < 0.01), as shown in the predicted line (slope: −109 245). Data displayed include all five participants with eight observations each (two language and two non-language regions per hemisphere). When creating the predicted lines, we used the left hemisphere, language regions, and the medians for age at death (64) and postmortem interval (14) for the adjusting variables.

We previously reported on asymmetry of NFT density and the language region selectivity of NFT and AP densities in this PPA-AD cohort (59). In this study, further analyses were conducted on neurons and microglia to evaluate concordance with AD neuropathology in PPA-AD. Neither NFTs nor dense-core APs were related to RM densities. NFT densities displayed positive relationships with gray matter HM densities (*P* < 0.01) and white matter activated microglia (*P* < 0.01), (Figure 5, Table S3). NFT densities also displayed a negative relationship with neuron densities (*P* = 0.01), (Figure 6, Table S3). Dense-core AP densities displayed negative relationships with HM densities (*P* < 0.01) and white matter activated microglia (*P* < 0.01), (Table S3). Dense-core AP densities also displayed a positive relationship with neuron densities (*P* < 0.01), (Table S3). Neuron densities were positively related to RM densities (*P* = 0.02), and inversely related to HM densities (*P* < 0.01) and white matter activated microglia (*P* < 0.01), (Figure 6, Table S3).

### Relationships between *in vivo* cortical atrophy and postmortem markers of microglia and neurons

**Figure 7.**
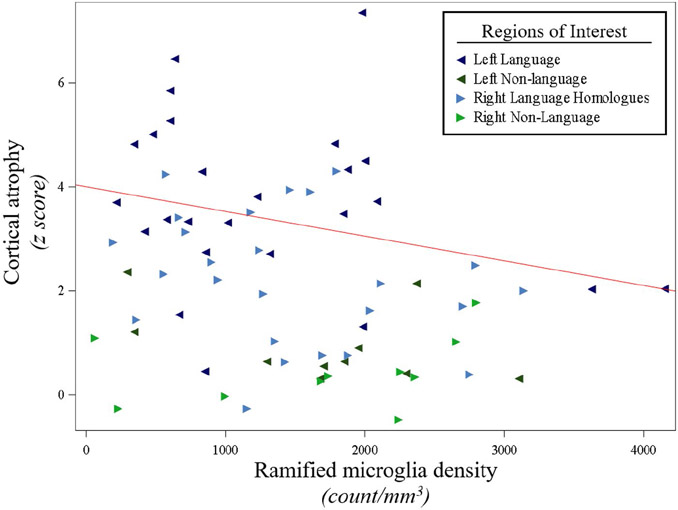
*Less* in vivo *cortical atrophy was associated with larger densities of ramified microglia in PPA-AD*. Cortical atrophy was negatively associated with ramified microglia densities (*P* = 0.03), as shown in the predicted line (−0.00047) from the mixed model, using the left hemisphere, language regions, and the medians for age at death (64) and scan-to-death interval (780) for the adjusting variables. Data displayed include all 5 participants with 14 observations each (five language and two non-language regions per hemisphere).

The current investigation found that cortical atrophy was not significantly associated with the densities of neurons, HM, or white matter activated microglia. However, greater cortical atrophy was related to lower densities of RM (*P* = 0.03), (Figure 7, Table S4).

## DISCUSSION

The cellular changes related to AD neuropathology and that contribute to cortical atrophy have received little attention in the non-amnestic clinical AD spectrum, especially PPA-AD. Here, we show that NFTs, but not APs, displayed positive relationships with activated microglia in the white and gray matter, providing new pathologic evidence of what may drive microglial activation and neuroinflammation in PPA-AD.

We also found that accumulations of both NFTs and activated microglia were associated with lower densities of neurons that likely reflect neurodegeneration in PPA-AD. However, the cellular basis of *in vivo* cortical atrophy remains incompletely understood in PPA-AD considering that neither neurons nor activated microglia were associated with MRI-based atrophy. These findings extend previous work by our group and others on the aphasic variant of AD (26, 50, 53, 59, 60, 67), which point to NFTs as a central determinant of microgliosis, neurodegeneration, and *in vivo* cortical atrophy in PPA-AD.

### Status of microglial activation and its relationship to neuropathology and cortical atrophy

Microglia exist in a dynamic surveillant state, ready to adopt diverse phenotypes that include activated microglia which can induce neuroinflammation. Our quantitative analysis of microglia suggests that HM and RM represent at least two discernible morphologic subtypes of HLA-DR-positive microglia (Figure 4). Converging evidence from human studies, animal models, and tissue culture suggest that HM are a widely observed morphologic subtype of activated microglia, while RM are commonly recognized as resting microglia (38, 55, 77, 79, 86). While future studies are needed to confirm these morphological subtypes of microglia and their functional properties in larger cohorts using other antibodies, we found that HM and RM display divergent distributions across the cortex in PPA-AD. Hypertrophic microglia were inversely related to RM and greatly outnumbered RM in all regions examined (Figure 4), possibly because of the HLA-DR antibody, a known marker for activated microglia (47), having a greater affinity for HM compared to RM. However, several investigations have reported that resting RM are also immunoreactive for HLA-DR but with lower intensity of staining (27, 48, 81), suggesting that the HLA-DR antibody recognizes a broad network of resident microglia that may constitutively express HLA-DR glycoproteins on RM (27) but become upregulated on HM in disease states (34, 47, 48). Furthermore, all tissue from our well-characterized PPA-AD cohort was processed similarly, with no known preagonal states that could impact the expression of HLA-DR as has been indicated previously (46). An alternative interpretation is that there may have been widespread activation and conversion of RM to HM in PPA-AD, which would be consistent with a previous report in amnestic AD indicating that most of the glial response is a phenotypic change as opposed to a proliferation of glia (74). The current study cannot speculate on the extent of glial proliferation in PPA-AD, but a phenotypic change from RM to HM may have resulted in a disproportionate increase in HM densities compared to RM densities in PPA-AD.

Consistent with a potential shift from RM to HM and their divergent roles in the PPA-AD brain, we found distinct associations between each microglial subtype and AD neuropathology. Hypertrophic microglia accumulated more in regions with less frequent neurons and more frequent NFTs and white matter activated microglia. In contrast, RM accumulated more in regions with fewer HM, more neurons, and less atrophy. The negative relationship between RM and cortical atrophy in PPA-AD (Figure 7) is similar to findings from a recent study that reported the loss of RM in severely atrophied inferior temporal regions of an AD cohort (61). Therefore, RM may act as benign sentinels not involved with neurodegenerative processes until they convert to HM in response to NFTs, which they were strongly associated with in PPA-AD. In fact, the positive association we found between NFTs and HM in PPA-AD is consistent with increasing evidence for a strong link between NFTs and activated microglia in non-amnestic presentations of AD (9) and in amnestic AD (55, 75). HM were also negatively related to dense-core APs which was surprising given that activated microglia have been positively associated with dense-core APs in amnestic AD (36, 75, 76). Resolving these discrepant findings may require exploring other intermediate forms of APs and microglia given that each of their dynamic morphologies have been previously shown to be linked (77).

Gray and white matter relationships are rarely examined postmortem in neurodegenerative diseases. Here, we found novel evidence that neurodegenerative processes may not be restricted to the gray matter in PPA-AD given that microglial activation was consistently elevated in the gray matter and adjacent white matter of atrophied language areas relative to non-atrophied non-language regions. Axonal degeneration and myelin loss have been known to occur in amnestic AD (19, 78) and similar events are likely to occur in PPA (14, 25, 54). If so, the recruitment of microglia would be likely as they are the primary phagocytes of the brain that activate and clear the axonal and myelin debris resulting from this degeneration. Wallerian degeneration may have led to these white matter changes in PPA-AD given that NFT accumulation in gray matter was associated with the microglial activation in both gray and white matter (Figure 5). In parallel, retrograde Wallerian-like degeneration is also conceivable given that tau has an endogenous axonal prominence (8) and its pathologic hyperphosphorylated state disrupts microtubule stability (18) that compromises axonal transport and thereby white matter integrity (6, 80). Therefore, the association between NFTs and widespread activated microglia in PPA-AD may reflect a leading neurodegenerative mechanism centered on NFTs and microglia-mediated neuroinflammation. However, amyloid-β oligomers and fibrils cannot be excluded from these pathological events given the evidence of their independent and tau-dependent neurotoxicities (13, 42, 45). Amyloid-β peptides are also known to cause microglia-mediated inflammation (7) and have been found in greater concentrations in the white matter compared to gray matter in amnestic AD (16). More research is needed to identify what aspects of white matter are altered and to what extent pathologic tau and amyloid-β promote white matter neuroinflammation in PPA-AD.

Unlike cortical atrophy patterns, densities of microglial subtypes did not display significant hemispheric or language region selectivity. This was surprising given that HM were positively related to the highly asymmetric NFT deposits and that activated microglia have been shown to be asymmetric in PPA with underlying TDP-43 pathology (39). However, it is possible that during the short timeframe between last MRI scan and death, the rate of microglial accumulation or phenotypic change could have plateaued or diverged from the rate of atrophy in each hemisphere. In fact, a recent PET study demonstrated that microglia may have an early and late phase of activation over the course of disease progression of AD (21). Greater mean densities of HM were observed in left language regions compared to right homologues, but regional asymmetry was subtle and inconsistent in each PPA-AD participant. While HLA-DR was useful to this investigation because of its reliable detection of both activated microglia (47, 48) and resting microglia (27, 81), other markers of activated microglia (34) or reactive subtypes of microglia may display more asymmetric patterns related to the PPA profile.

### Neuron densities reflect selective neurodegeneration

Lower neuron densities likely reflect neurodegeneration given their association with higher densities of NFTs and HM in the same regions (Figure 6). However, similar to distributions of HM, mean densities of neurons were not significantly lower in left language regions compared to right homologues. Neuron densities were not related to *in vivo* cortical atrophy, possibly because of MRI scans not capturing neurodegeneration that continued after the final scan and before death. Alternatively, the quantification of NeuN-positive neurons may have been a limitation given that NeuN immunoreactivity has been shown to decline with disease severity suggesting that NeuN might be a better marker of neuronal health compared to neuron loss (89). Therefore, the fairly symmetric patterns of NeuN-positive neuron densities in PPA-AD might represent a general state of poor neuronal health across the cortex by end stage of disease that is not directly reflective of the significant cortical atrophy potentially mediated by neuron loss in PPA.

The cellular basis of *in vivo* cortical atrophy remains mostly unknown, but select studies that examined unilateral hippocampi in amnestic AD brains have reported that hippocampal CA1 neuron number correlated with cortical atrophy (41, 90). In contrast, but consistent with our findings in PPA-AD, several other studies have shown that neuron densities (20, 24, 32) and activated microglia densities (75) do not correlate with postmortem or antemortem measurements of cortical thickness. It is possible that other neuronal changes are more sensitive to pathologic alterations and should be explored to expand our understanding of the cellular basis of neurodegeneration. Indeed, changes in components of cells (i.e., synapses, dendrites, and somas), vasculature, or other cellular populations may in some combination make more significant contributions to cortical atrophy than NeuN-positive neurons or HLA-DR-positive microglia.

The reliable identification of the potential neuropathologic and cellular determinants of *in vivo* cortical atrophy in the focal, asymmetric profile of PPA required a stereologic investigation of bilateral hemispheres in PPA-AD participants with MRI scans close to death. However, this strict inclusion criteria and rigorous design in a relatively rare clinical population resulted in a small cohort that were all male. Novel quantitative studies involving larger PPA cohorts with balanced sexes will be needed to help resolve which neurons and glia are most vulnerable to AD neuropathology and likely contribute to cortical atrophy in the aphasic variant of AD.

## CONCLUSIONS

In summary, the current investigation showed that microglial subtypes are distinguishable by morphology and may have differential involvement in neurodegeneration based on their unique associations with densities of neurons and AD neuropathology in PPA-AD. Only NFT density was associated with greater densities of HM and white matter activated microglia, suggesting that the microglial response may be more sensitive to NFTs compared to APs and other neurodegenerative processes. Upon activation, microglia may have mediated deleterious inflammation that worsened the disease process in both the gray and white matter in PPA-AD. Therefore, the smaller densities of neurons associated with larger densities of NFTs and activated microglia suggest that multiple pathologic and inflammatory markers drive patterns of focal neurodegeneration characteristic of PPA-AD.

## Supplementary Material

Supplement **Table S1.** Hemispheric and language region selectivity of microglial subtypes, adjusted for age at death and postmortem interval.
**Table S2.** Hemispheric and language region selectivity of neurons, adjusted for age at death and postmortem interval.
**Table S3.** Relationships between postmortem variables, adjusted for hemisphere, type of region, age at death, and postmortem interval.
**Table S4.** Relationships between cortical atrophy and postmortem variables, adjusted for hemisphere, type of region, age at death, and scan-to-death interval.

## Figures and Tables

**Table 1.** Characteristics of PPA-AD participants.

PPA-AD participant #	PPA sub-type	Sex	Education (years)	Age at onset (years)	Age at scan (years)	Age at death (years)	Symptom duration (years)	Scan/death interval (years)	Postmortem interval (hours)	Fixative	*APOE* status
1	L	Male	16	56	61	62	4.7	0.6	6	F	*3,3*
2	U	Male	12	55	61	64	7.9	2.1	14	F	*3,3*
3	L	Male	15	67	73	76	8.6	2.0	8	P	*3,4*
4	U	Male	14	51	58	61	9.4	2.5	19	P	*3,4*
5	G	Male	18	72	75	78	6.2	2.5	28	P	*3,4*

Ages were calculated from date of birth to event (i.e., disease onset, scan, or death) and were rounded for summary purposes.

Abbreviations: *APOE* = apolipoprotein E; F = formalin; G = agrammatic; L = logopenic; P = paraformaldehyde; U = unclassifiable due to severity of impairments.

**Table 2.** Subset of regions used in analyses of white matter activated microglia.

PPA-AD participant #	1	2	3	4	5
Non-language	V1	V1	V1	V1	V1
	EC	EC	EC	EC	EC
Language	aIPL	IFG	pSTG	pSTG	aSTG
	pIPL	aSTG	aIPL	aIPL	pSTG

Language regions were chosen based on their relatively high degree of cortical atrophy in the language-dominant hemisphere compared to contralateral homologues and non-language regions.

Abbreviations: aIPL = anterior inferior parietal lobule; aSTG = anterior superior temporal gyrus; EC = entorhinal cortex; IFG = inferior frontal gyrus; pIPL = posterior inferior parietal lobule; pSTG = posterior superior temporal gyrus; V1 = primary visual cortex.

## Data Availability

The data sets during and/or analyzed during the current study are available from the corresponding author on reasonable request.
