# Supplementary material for: Accumulation of neurofibrillary tangles and activated microglia is associated with lower neuron densities in the aphasic variant of Alzheimer’s disease
Source: Brain Pathol. 2020 Nov 5;31(1):189–204. doi: 10.1111/bpa.12902 (PMC7855834; doi:10.1111/bpa.12902)
Supplement: Supplementary file 1 — Table S1. Hemispheric and language region selectivity of microglial subtypes, adjusted for age at death and postmortem interval. Table S2. Hemispheric and language region selectivity of neurons, adjusted for age at death and postmortem interval. Table S3. Relationships between postmortem variables, adjusted for hemisphere, type of region, age at death, and postmortem interval. Table S4. Relationships between cortical atrophy and postmortem variables, adjusted for hemisphere, type of region, age at death, and scan‐to‐death interval. [file BPA-31-189-s001.docx]

**SUPPLEMENTARY TABLES**

***Table S1. Hemispheric and language region selectivity of microglial subtypes, adjusted for age at death and postmortem interval.***

Significance level set to α = 0.05. SE = standard error; HM = hypertrophic microglia; RM = ramified microglia.

| **Outcome** | **Stratified by** | **Covariate** | **Estimated Mean Densities (SE)** | | ***p*-value** |
| --- | --- | --- | --- | --- | --- |
|  |  |  | **LEFT** | **RIGHT** |  |
| HM | Language | Left/Right | 8988 (1834) | 7475 (1834) | 0.08 |
| HM | Non-language | Left/Right | 8604 (1876) | 7492 (1875) | 0.67 |
| RM | Language | Left/Right | 1309 (450) | 1461 (450) | 0.43 |
| RM | Non-language | Left/Right | 1690 (440) | 1703 (440) | 0.97 |
| **Outcome** | **Stratified by** | **Covariate** | **Estimated Mean Densities (SE)** | | ***p*-value** |
|  |  |  | **LANGUAGE** | **NON-LANGUAGE** |  |
| HM | Left | Language/Non-language | 8988 (2046) | 8604 (2214) | 0.78 |
| RM | Left | Language/Non-language | 1309 (449) | 1690 (481) | 0.22 |

***Table S2. Hemispheric and language region selectivity of neurons, adjusted for age at death and postmortem interval.***

Significance level set to α = 0.05. SE = standard error.

| **Outcome** | **Stratified by** | **Covariate** | **Estimated Mean Densities (SE)** | | ***p*-value** |
| --- | --- | --- | --- | --- | --- |
|  |  |  | **LEFT** | **RIGHT** |  |
| Neurons | Language | Left/Right | 30125 (3640) | 35164 (3640) | 0.10 |
| Neurons | Non-language | Left/Right | 25300 (4637) | 27519 (4637) | 0.80 |
| **Outcome** | **Stratified by** | **Covariate** | **Estimated Mean Densities (SE)** | | ***p*-value** |
|  |  |  | **LANGUAGE** | **NON-LANGUAGE** |  |
| Neurons | Left | Language/Non-language | 30125 (4214) | 25300 (4912) | 0.28 |

***Table S3. Relationships between postmortem variables, adjusted for hemisphere, type of region, age at death, and postmortem interval.***

All analyses included 14 regions per participant (N=70 total regions), except for analyses of white matter activated microglia (WMAM) which included 8 regions per participant (N=40 total regions). Significance level set to α = 0.025. SE = standard error; NFT = neurofibrillary tangles; AP = amyloid-ß plaque; HM = hypertrophic microglia; RM = ramified microglia; WMAM = white matter activated microglia.

| **Outcome** | **Covariate** | **Parameter Estimates (SE)** | | **P value** |
| --- | --- | --- | --- | --- |
| NFT | HM | 0.250 | (0.072) | *p*<0.01 |
| NFT | RM | -0.612 | (0.391) | *p*=0.12 |
| NFT | Neurons | -0.057 | (0.022) | *p=*0.01 |
| NFT | WMAM | 18212 | (4846) | *p*<0.01 |
| AP | NFT | -0.013 | (0.021) | *p*=0.55 |
| AP | WMAM | -4000 | (834) | *p*<0.01 |
| HM | AP | -3.816 | (1.043) | *p*<0.01 |
| HM | Neurons | -0.129 | (0.033) | *p*<0.01 |
| HM | WMAM | 54457 | (8013) | *p*<0.01 |
| RM | AP | 0.290 | (0.216) | *p*=0.18 |
| RM | HM | -0.085 | (0.022) | *p*<0.01 |
| RM | Neurons | 0.016 | (0.007) | *p=*0.02 |
| RM | WMAM | -5885 | (1213) | *p*<0.01 |
| Neurons | AP | 11.327 | (3.677) | *p*<0.01 |
| Neurons | WMAM | -109245 | (34237) | *p*<0.01 |

***Table S4. Relationships between cortical atrophy and postmortem variables, adjusted for hemisphere, type of region, age at death, and scan-to-death interval.***

Significance level set to α = 0.05. SE = standard error; HM = hypertrophic microglia; RM = ramified microglia; WMAM = white matter activated microglia.

| **Outcome** | **Covariate** | **Parameter Estimates (SE)** | | **P value** |
| --- | --- | --- | --- | --- |
| Atrophy | HM | 0.00003 | (0.00004) | *p*=0.48 |
| Atrophy | RM | -0.0005 | (0.0002) | *p=*0.03 |
| Atrophy | Neurons | -0.000006 | (0.00001) | *p*=0.65 |
| Atrophy | WMAM | 3.653 | (2.583) | *p*=0.17 |
